# Studying Memory Encoding to Promote Reliable Engagement of the Medial Temporal Lobe at the Single-Subject Level

**DOI:** 10.1371/journal.pone.0119159

**Published:** 2015-03-24

**Authors:** Marta Simó, Pablo Ripollés, Lluís Fuentemilla, Lucía Vaquero, Jordi Bruna, Antoni Rodríguez-Fornells

**Affiliations:** 1 Cognition and Brain Plasticity Group, Bellvitge Biomedical Research Institute-IDIBELL, L’Hospitalet de Llobregat, Barcelona, Spain; 2 Neuro-Oncology Unit, Hospital Universitari de Bellvitge (HUB) and Hospital Duran i Reynals (Institut Català d’Oncologia), L’Hospitalet de Llobregat, Barcelona, Spain; 3 Department of Basic Psychology, Bellvitge Campus, University of Barcelona, L’Hospitalet de Llobregat, Barcelona, Spain; 4 Catalan Institution for Research and Advanced Studies, ICREA, Barcelona, Spain; Zhejiang Key Laborotory for Research in Assesment of Cognitive Impairments, CHINA

## Abstract

The medial temporal lobe (MTL)—comprising hippocampus and the surrounding neocortical regions—is a targeted brain area sensitive to several neurological diseases. Although functional magnetic resonance imaging (fMRI) has been widely used to assess brain functional abnormalities, detecting MTL activation has been technically challenging. The aim of our study was to provide an fMRI paradigm that reliably activates MTL regions at the individual level, thus providing a useful tool for future research in clinical memory-related studies. Twenty young healthy adults underwent an event-related fMRI study consisting of three encoding conditions: word-pairs, face-name associations and complex visual scenes. A region-of-interest analysis at the individual level comparing novel and repeated stimuli independently for each task was performed. The results of this analysis yielded activations in the hippocampal and parahippocampal regions in most of the participants. Specifically, 95% and 100% of participants showed significant activations in the left hippocampus during the face-name encoding and in the right parahippocampus, respectively, during scene encoding. Additionally, a whole brain analysis, also comparing novel versus repeated stimuli at the group level, showed mainly left frontal activation during the word task. In this group analysis, the face-name association engaged the HP and fusiform gyri bilaterally, along with the left inferior frontal gyrus, and the complex visual scenes activated mainly the parahippocampus and hippocampus bilaterally. In sum, our task design represents a rapid and reliable manner to study and explore MTL activity at the individual level, thus providing a useful tool for future research in clinical memory-related fMRI studies.

## Introduction

Functional magnetic resonance imaging (fMRI)—a non-invasive method that enables mapping of neural activity [1]—has been widely used to assess brain functional abnormalities in numerous studies dealing with neurological diseases [1–3]. However, one important limitation of this technique is that brain activity depends significantly on the behavioral paradigm employed. An effective fMRI paradigm allows enhancing the activity of a targeted brain structure during the course of a specific cognitive behavior. Hence, the choice of the type of stimulus (e.g. verbal or nonverbal), the type of mnemonic process (e.g. item versus recognition of associative relations) and appropriate comparison across conditions (e.g. novel versus repeated stimulus) is critical for the detection and discrimination of a set of targeted brain areas thought to be involved in a particular cognitive process (e.g. episodic memory) [4].

The medial temporal lobe (MTL) comprises the hippocampus (HP) and the surrounding neocortical regions including the parahippocampal gyrus (PHG), comprised of the entorhinal and the perirhinal cortex as well as the posterior parahippocampal cortices. It is widely agreed that the MTL plays a critical role in memory across species [5–7], although the fundamental mechanism by which the MTL accomplishes this remains subject to debate. Within the MTL, the HP sits in a central position where most neural computation regarding memory processes seems to be governed. Thus, it has long been suggested that the unique anatomical circuitry of the HP makes it ideally suited to act as a comparator, identifying discrepancies between current sensory reality and past experience [8,9] and also in associative processing within the spatial and non-spatial domains [10]. As a result, the HP has been established as a key area to detect the novel associative elements unfolding before us [8,9]. Interestingly, it has also been shown that such novelty detection is further supported by a distributed brain network outside the HP, including other MTL structures such as the PHG and neocortical regions such as the lateral and orbital prefrontal cortex, as well as the anterior temporal and temporo-parietal cortices [9,11,12].

Given the important role of HP in novelty detection and its interest as a targeted brain region sensitive to several neurological diseases [1], the primary goal of the current research was to provide an experimental approach allowing reliable identification, at the individual level, of HP activity induced by novelty detection. Interestingly, in spite of HP activation’s being a hallmark in the clinical setting, it has proven to be technically challenging due to its great susceptibility to signal loss [13,14]. This artifact results from abrupt changes in macroscopic field gradients occurring in air-tissue or at bone boundaries, especially in whole-brain analysis [15]. Here, as suggested previously [16,17], we implemented a region-of-interest (ROI) approach which allows a more quantitative analysis that can focus on statistical comparisons among MTL subregions.

In addition, the current study also examined to what extent associative novelty processing in the HP was material-dependent. And although previous research leads to the proposal that HP novelty detection is not domain-specific [3,18–24], this has not been systematically scrutinized at the individual level.

To address these issues, we used an event-related fMRI paradigm in healthy individuals that, in combination with the aforementioned ROI-based analysis, tested the hypothesis that HP novelty signals can be reliably detected at the individual level and independently of the experimental material, thereby opening the door to their use in future clinical scenarios.

## Methods

### Participants

Twenty healthy young adults between 20 and 32 years of age (13 women, mean age 26.15 ± 3.26 years) were recruited from the University of Barcelona—Hospital of Bellvitge campus. All participants were right-handed, native Spanish speakers and had a similar level of education (20 ± 1 year). None of them reported a history of psychiatric or neurological disorder, or psychoactive drug use. The study was approved by the local Hospital of Bellvitge Research Ethics Board, and each participant gave written informed consent.

### Stimuli

Stimuli used in the current experiment comprised a set of 121 pairs of words (W), 122 black and white pictures of faces that were presented in association with 122 common Spanish names (F) and 120 black and white pictures of scenes (S). Words were middle frequency abstract nouns selected from previous word experiments [1]. Although the three types of stimuli (W,F,S) were not matched in familiarity or frequency among them, we ensured that the selected abstract words were highly familiar, and low in imageability and concreteness (mean familiarity: 5.9; mean imageability: 3.3; mean concreteness: 3.6; rated on scales ranging from 1-low to 7-high) [1]. A set of human faces was selected from different United States face databases. Sixty-one were female and sixty-one male. Faces were presented together with high frequency Spanish names selected from the Spanish National Institute of Statistics (INE, Instituto Español de Estadística). Complex visual scenes were selected from online scene databases. Sixty pictures were indoor scenes and sixty were outdoor scenes. One abstract black and white noise image (containing the same luminosity and colour as one of the presented images, but degraded) was selected for the baseline condition, as this has been proven to be a good control condition to elicit MTL activation when using complex scenes [25].

### Experimental Design

**Fig 1:**
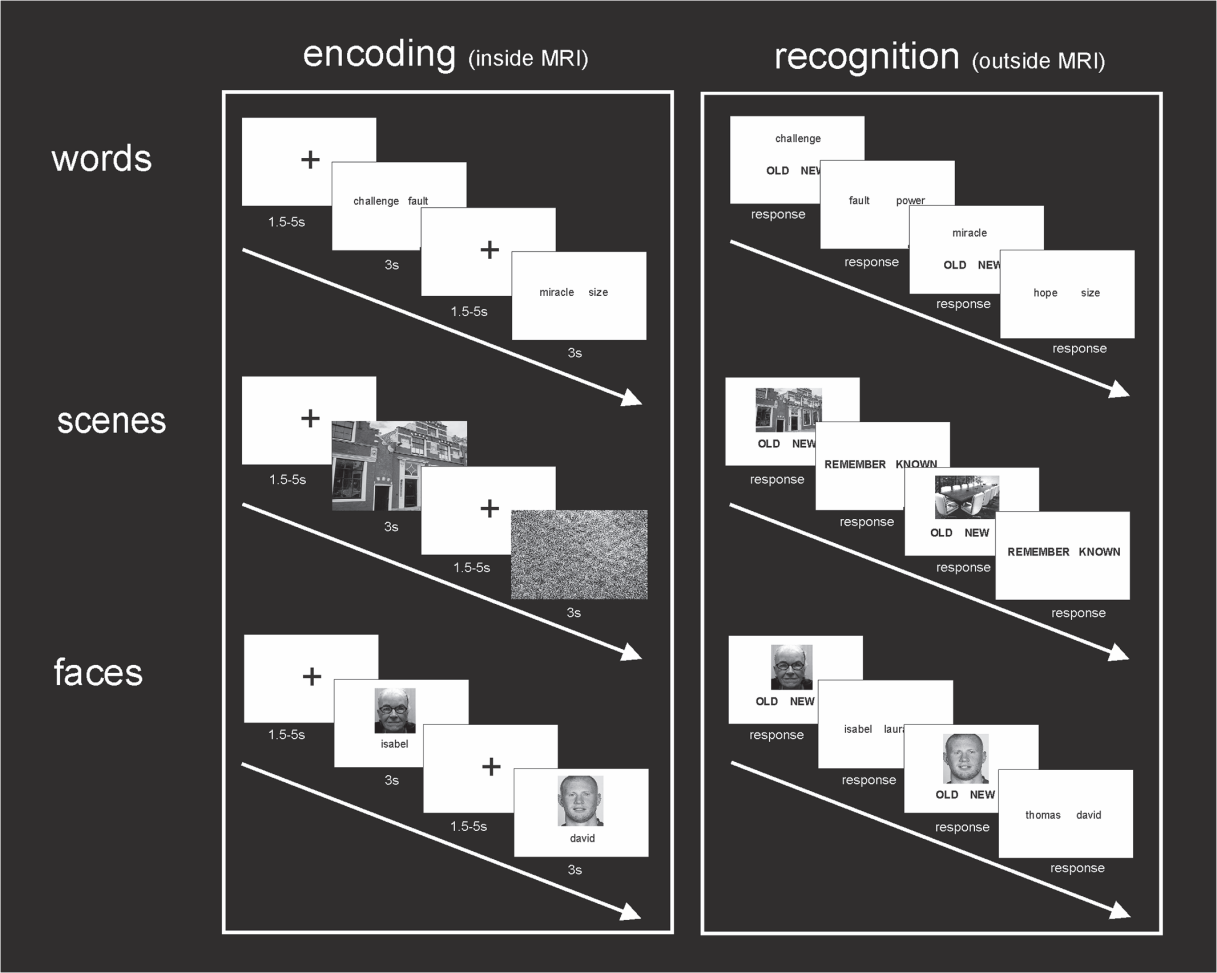
Illustration of the experimental task design. Encoding and recognition tests. Each encoding run inside the MRI scanner contained only one type of novel and repeated stimuli material. The recognition test outside the MRI scanner was performed following the same order of presentation.

The experimental task included an encoding and a testing phase (Fig. 1). The encoding phase was performed inside the MRI scanner and was divided into 3 runs. Each run contained only one of the three different types of stimuli to be encoded (W, F, S). The order of presentation of the encoding runs (W, F, S) was counterbalanced across participants and the order of presentation of novel and repeated trials within each run was randomized. We ensured that no more than two repeated trials were consecutively presented and that at least four repeated trials appeared each 15 trials. Participants were informed that a memory test would follow the encoding phase. Total MRI scanning time was 30 min. The recognition test was administered at the end of the encoding task outside the MRI scanner and was divided in 3 parts. Each part consisted of only one of the three types of encoding information performed previously during the fMRI task (W, F, S). For each subject, the order of the parts during testing followed the one used during the encoding phase.

#### Encoding phase

There were three encoding scanning runs comprising 90 trials each (60 novel trials and one trial repeated 30 times, see Fig. 1 for further details). Study items (trials) were centrally presented for 3 seconds followed by an interval of 1.5–5 seconds (central fixation cross, 90 trials). In all cases, some stimuli were presented once (hereafter referred to as ‘novel’) and others were shown repeatedly (hereafter referred to as ‘repeated’).

During the word-pair association condition, participants were instructed to silently generate a sentence using both words during novel trials (60 pairs of words) [26]. In repeated trials (one pair repeated 30 times), participants were requested to generate a sentence by themselves, and to use this same sentence each time [19]. Three word-word pairs were randomly selected to be used as the repeated stimuli. Therefore, three different versions of the word-pair task (the only difference among them was the pair of words being used as the repeated stimuli) were presented, counterbalanced across participants.

Novel trials in the face-name association condition included 60 face-name pairs, 30 female and 30 male. Repeated trials included 2 face-name pairs (1 female and 1 male) shown 15 times each [27]. In this case, three sets of 2 face-name pairs (1 female and 1 male) were selected as repeated stimuli. Again, three different versions of the face-name experiment were presented, also counterbalanced across subjects. The only difference among the three versions of the experiment was the set of 2 face-name pairs being used as the repeated stimuli.

In the complex visual scene condition, participants were instructed to report whether the scene stimuli were indoor or outdoor images during novel trials but not during the repeated image. Participants were instructed to fix their gaze on the centre of the image during both conditions. This novel condition included 30 indoor and 30 outdoor scene pictures. The repeated condition consisted of a single noise image that was presented 30 times [25].

#### Recognition phase

The recognition test was performed outside the scanner. Each of the recognition parts of the test, one for each type of encoded material, consisted of 120 trials: 60 trials corresponded with trials encoded as novel during the encoding phase (‘Old’ items) and the other 60 trials corresponded with items not seen during the encoding phase (‘New’ items). In all conditions, the trial remained on the screen until participants made an ‘Old/New’ distinction through button press. Items that were correctly recognized (participants answered correctly ‘Old’) were identified as ‘Hits’. Items that were not recognized (participants incorrectly answered that the item was 'New') were identified as incorrect rejections. ‘Old’ responses were followed by a two-alternative forced-choice task in which participants were required to choose which of the two items appearing on the screen corresponded to the item presented during the encoding phase.

In the *word pair condition*, ‘Old’ words consisted always of the one presented on the left side of the screen during the encoding phase. ‘Old’ responses were followed by the presentation of two words. Participants were instructed to indicate which of the two words was correctly associated with the previous one during the encoding phase (both words had appeared inside the scanner but in different pairs). In the *face-name condition*, a face appeared on the screen (in half of the trials the face was female and in the other half male). ‘Old’ responses were followed by the presentation of two names, and participants were instructed to indicate the correct name associated with that particular face during the encoding phase (both names had appeared inside the scanner but in different pairs). During this recognition test, the provided incorrect words and face names belong to those used during the encoding phase. To control for possible familiarity-based effects, accounted for by the fact that an item was presented more than once in the recognition phase, we set the number of appearances of each item at two, ensuring that all items were presented once as a correct and another time as an incorrect response. In the *scene condition*, a picture scene appeared on the screen. ‘Old’ responses were followed by a ‘Remember/Know’ task [14]. Participants were instructed to select ‘Remember’ if their memory of that scene was accompanied by a vivid detailed recollection of the episode in which the image was embedded. Alternatively, participants had to select ‘Know’ if the scene only aroused a feeling of familiarity.

### fMRI acquisition

Scanning was performed on a 3-T Siemens Trio System. Functional data were acquired using a gradient echo pulse sequence (32 transverse slices oriented along the anterior-posterior commissural axis with a 30-degree upward tilt to avoid the eyes, repetition time of 2 s, echo time of 30 ms, 3 x 3 x 3.5 mm voxels, 0.8-mm interslice gap). A high-resolution T1-weighted magnetization-prepared rapid acquisition gradient echo (MPRAGE) image (240 slices sagittal, TR = 2300 ms, TE = 2.98 ms, 1 mm isotropic voxels) was also collected.

### fMRI analysis

fMRI data were analyzed using standard procedures implemented in the Statistical Parametric Mapping software (SPM8, Wellcome Trust Centre for Neuroimaging, University College, London, UK, www.fil.ion.ucl.ac.uk/spm/). Preprocessing consisted of several steps. Images were first corrected for differences in slice timing acquisition using the middle slice of each volume as the reference, and then spatially realigned with respect to the first volume of the first run. Each participant’s MPRAGE scan was co-registered to the mean echo planar imaging (EPI) volume, produced in the previous step during spatial realignment. Each coregistered structural scan was then segmented using New Segment [28] and normalized with diffeomorphic anatomical registration through exponentiated lie algebra (DARTEL) SPM8 toolbox [29]. DARTEL is a suite of tools fully integrated with SPM8 which has been shown to achieve optimal normalization in healthy subjects [30,31]. This method also achieves better localization of fMRI activations in Montreal Neurological Institute (MNI) space, and it has been successfully applied in several previous studies dealing with both healthy and patient populations [32]. Briefly, the grey and white matter segmented images obtained were used to create a study specific template using the DARTEL toolbox. Then the flow fields containing the deformation parameters to this template were used to normalize each participant’s realigned EPIs to MNI space. Finally, normalized EPI images were re-sliced to 2 x 2 x 2 mm and smoothed with an 8 mm full-width at half-maximum isotropic Gaussian kernel.

First-level statistical analysis was based on a least square estimation using the general linear model. The different conditions were modelled with a regressor waveform convolved with a canonical hemodynamic response function. For each individual subject, statistical parametric maps (SPMs) were generated. Specifically, an event-related design matrix was created for each subject, which included *novel*, *repeated* and *fixation* (central fixation cross) conditions for all three stimuli (W, F, S). The movement parameters (estimated during the realignment phase) were also included in the model to correct for motion effects. Finally, contrast images for each of the main conditions (novel, repeated and fixation) were calculated for each subject. Group activation was calculated using a random effects model. The individual main contrast images for W, F and S were entered into a second level repeated measures ANOVA to test for the ‘Novelty effect’ (novel relative to repeated) generated by the three types of stimuli altogether. Paired t-tests were also calculated to analyze the novelty effect of each type of stimuli (W, F, S). In order to visually explore the common regions activated by each stimulus type (W, F, S) activation masks for the novelty effect were calculated at the selected threshold (see below) and an overlap image was created. Finally, to assess the differences in novelty between types of stimuli, we calculated several 2 (Condition) x 2 (Type of stimulus) interactions: Novelty—Repeated Words > Novelty—Repeated Faces, Novelty—Repeated Words > Novelty—Repeated Scenes, Novelty—Repeated Faces > Novelty—Repeated Scenes and their reversed contrasts. All results are reported at false discovery rate (FDR) corrected p < 0.05 threshold at the voxel level with a cluster extent of 50 contiguous voxels.

#### ROI analysis: HP and PHG

We investigated the degree to which HP activity was reliably identified at the individual level with our experimental design. We also extended this approach to the PHG, known to play an important supporting role during successful memory encoding [2]. Thus HP and PHG ROIs were defined based on the Anatomical Automatic Labelling Atlas [33] in MNI space using the WFU pickatlas tool [34,35]. Then, three material specific contrasts (novelty versus repeated words; novelty versus repeated faces; novelty versus repeated scenes) and one general contrast (novelty versus repeated words & faces & scenes) were calculated for each participant at the individual level. The defined ROIs were applied to these functional contrasts in order to quantify the percentage of subjects activating left, right or bilateral HP, or PHG in response to novelty. As in previous fMRI studies [16,36–40], we adopted a threshold approach, based on the number of contiguous activated voxels, to assess activation in the hippocampus. In the current study, the significance threshold was set at p < 0.05 (uncorrected) with a minimum of 5 adjacent activated voxels within each ROI. We therefore report voxels with a peak value of activation of at least p < 0.05 and that form part of a cluster of at least 5 contiguous voxels [16]. The selected clusters of voxels were binarized and added together to show the spatial extent of HP and PHG activation in our sample. Moreover, the number of activated voxels in the left and right hemispheric ROIs were compared, using a paired t-test, to account for material-dependent laterality effects at encoding.

## Results

### Behavioural results

Results from behavioural data are detailed in Table 1. Participants performed above chance with an overall hit rate (including the 3 conditions) of 58 ± 13%. Of these overall correctly recognized items, participants correctly associated faces with names and word pairs in 79 ± 12%. This probably means that while participants had difficulties in remembering all the stimuli, they created strong associations for the stimuli that they actually remembered. Participants showed similar memory accuracy during the recognition test for all three conditions (F (2, 38) = 1.80, p > 0.18).

**Table 1:** Mean rate of hits, false alarms, correct hits and correct rejections with standard deviation in parentheses.

Performance	Words	Scenes	Faces
Hits	0.61 (0.15)	0.55 (0.14)	0.56 (0.15)
Correct rejection	0.76 (0.11)	0.80 (0.08)	0.88 (0.07)
False alarm	0.24 (0.11)	0.20 (0.08)	0.10 (0.07)
Correct association	0.85 (0.12)	-	0.72 (0.14)
Remembered	-	0.68 (0.13)	-

*Hits and correct rejections*: items that were correctly recognized in the testing phase (participants answered correctly ‘Old’ or ‘New’, respectively). *False alarm*: items that were incorrectly recognized in the testing phase as ‘Old’. *Correct association*: items that were correctly associated in the two alternative forced-choice task in the words and faces conditions. *Remembered*: items of the scenes condition that were identified as remembered in the ‘Remember/Know’ task.

### Hippocampal ROI analysis

**Fig 2:**
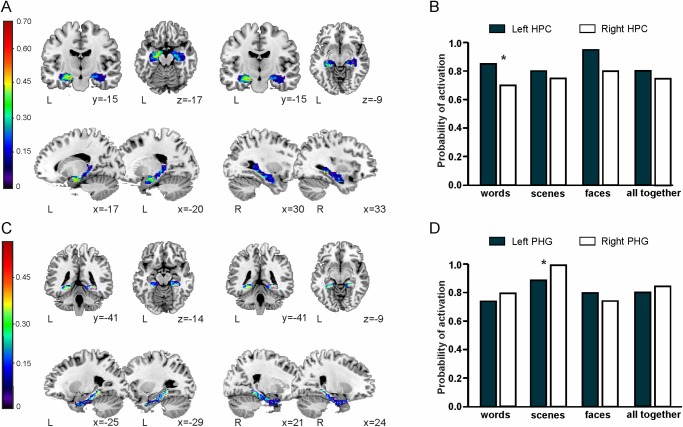
MTL ROI analysis. *A*. Spatial distribution of HP activations at the individual level. Each voxel represents the percentage of subjects in which a novelty effect was found in any of the three encoding conditions (words, faces, scenes) at the individual level (in a particular subject a voxel is counted as activated if any of the three types of stimuli yields an activation above the selected p < 0.05 threshold with 5 voxels of spatial extent). *B*. Percentage of participants that showed significant (p < 0.05 uncorrected at the subject level) novelty-related activations in any group of at least 5 contiguous voxels inside the HP ROI. The percentages are reported for each stimulus separated (words, faces, scenes) and for all stimuli pooled together. *C*. Spatial distribution of PHG activations at the individual level. Each voxel represents the percentage of subjects in which a novelty effect was found in any of the three encoding conditions (words, faces, scenes) at the individual level (in a particular subject a voxel is counted as activated if any of the three types of stimuli yields an activation above the selected p < 0.05 threshold with 5 voxels of spatial extent). *D*. Percentage of participants that showed significant (p < 0.05 uncorrected at the subject level) novelty-related activations in any group of at least 5 contiguous voxels inside the PHG ROI. The percentages are reported for each stimulus separated (words, faces, scenes) and for all stimuli pooled together. Colour-coded scale indicates the percentage of participants that showed activation at the individual level in a particular voxel in any of the three tasks. Spatial maps are overlaid over a canonical template with MNI coordinates at the bottom right of each slice. * Significant (p < 0.05) difference in amount of voxels activated between the left and right ROI (see Methods and Results).

Overall, more than 70% of the sample showed HP activation bilaterally when the three encoding conditions were analysed together (mean activated voxels on left HP = 250.55; mean activated voxels on right HP = 211.55; non-significant differences in number of voxels between hemispheres, p > 0.59). When the analysis was performed on data from each encoding condition independently, only the *word pair condition* activated more voxels in the left than the right HP (85% of subjects activated left—mean activated voxels 205.95—and 70% right HP—mean activated voxels 91.45; p < 0.04). Both the *face-name condition* (90% of subjects activated left—mean activated voxels 250.55–80% activated right HP—mean activated voxels 211.65; p > 0.59) and the *complex visual scenes condition* (80% of subjects activated left—mean activated voxels 158.4—and 75% activated right HP—mean activated voxels 144.15; p > 0.76) activated the HP bilaterally in a similar manner. Fig. 2A displays the spatial distribution and Fig. 2B the percentage of participants showing activation in the HP. Specifically, Fig. 2A, which shows voxels in which at least one of the three conditions elicited significant activity, exhibits a predominantly left anterior HP activation.

### Parahippocampal ROI analysis

Up to 80% of subjects activated the PHG bilaterally when the three tasks were analysed together (mean activated voxels on left PHG = 174.25; mean activated voxels on right PHG = 253.6; non-significant differences in number of voxels between hemispheres, p > 0.15). Again, stimulus dependent differences were found. Specifically, both *words* (75% of subjects activated left—mean activated voxels 53.5—and 80% right—mean activated voxels 55.65; p > 0.87) and *face-names* (80% activated left—mean activated voxels 65.05–75% right—mean activated voxels 68.75; p > 0.45) yielded robust bilateral PHG activations. The *complex visual scenes condition*, however, activated more voxels in the right than in the left PHG (90% of subjects activated left, 100% activated right PHG; mean activated voxels on left = 92.7, and on right = 126.15; p < 0.002). Interestingly, while the *word-pair* and *face-name* conditions activated more anterior regions of the PHG—including the entorhinal and perirhinal cortex—the *complex visual scenes* task activated more posterior regions of the PHG (see Fig. 2C, the higher number of subjects engaging the posterior part of the PHG is driven by the scenes condition). Fig. 2C displays the spatial distribution and Fig. 2D the percentage of participants showing activation in the PHG.

**Fig 3:**
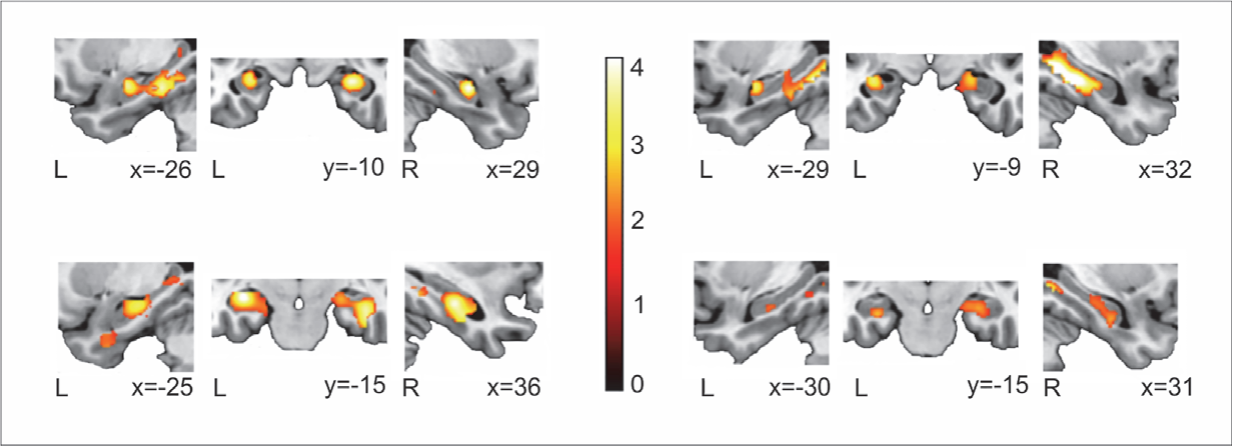
Individual examples for MTL activation at the subject level. In red-yellow, for four different participants, enhanced fMRI-signals for the Novel > Repeated encoding trials for all three stimuli pooled together (words, faces, scenes) using an ROI analysis centered in the hippocampus and parahippocampus. Neurological convention is used. All statistical maps are thresholded at p < 0.05 uncorrected threshold with 5 voxels of cluster extent and overlaid over a canonical template with MNI coordinates at the bottom right of each slice; L, Left Hemisphere; R, Right Hemisphere.

As an example, Fig. 3 shows, in four individuals, fMRI enhanced activity for the Novel > Repeated encoding trials for all three stimuli pooled together (words, faces, scenes) using a ROI analysis centred in the hippocampus and parahippocampus (p<0.05 uncorrected threshold, 5 voxels of spatial extent).

### Whole brain analysis: the novelty effect

**Fig 4:**
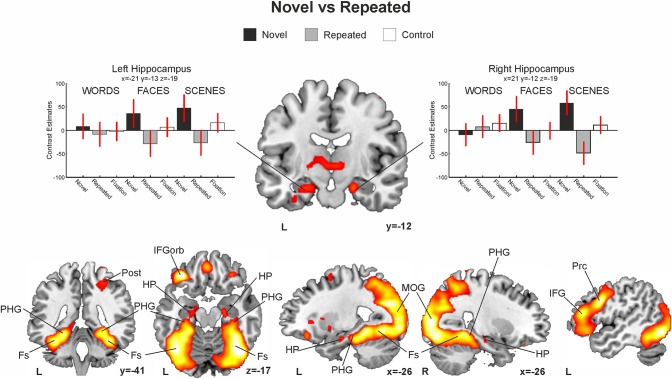
Whole brain novelty contrast. In red-yellow, enhanced group-level fMRI-signal for the Novel > Repeated encoding trials for all three stimuli pooled together (words, faces, scenes). Bar graphs on the right indicate contrast estimates (proportional to percent signal change) with 90% confidence intervals for the maximum peak in the left and right hippocampus (black for novel, grey for repeated, white for fixation). Neurological convention is used. All statistical maps are thresholded at p < 0.05 FDR-corrected with 50 voxels of cluster extent and overlaid over a canonical template with MNI coordinates at the bottom right of each slice. HP, hippocampus; PHG, parahippocampal gyrus; Fs, fusiform gyrus; Post, postcentral gyrus; IFGorb, inferior frontal gyrus pars orbitalis; MOG, middle occipital gyrus; Prc, precentral gyrus; IFG, inferior frontal gyrus; ITG, inferior temporal gyrus; L, Left Hemisphere; R, Right Hemisphere.

**Fig 5:**
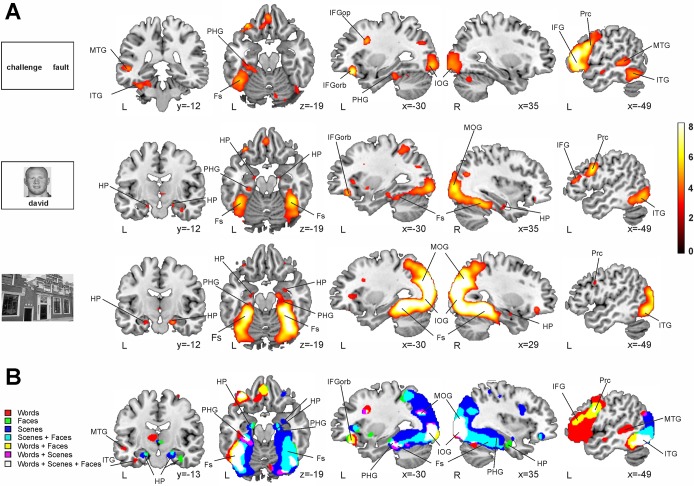
Whole brain material dependent novelty contrast. *A*. In red-yellow, enhanced group-level fMRI-signal for the Novel > Repeated encoding trials for words (top), faces (middle) and scenes (bottom). All statistical maps are thresholded at p < 0.05 FDR-corrected with 50 voxels of cluster extent. *B*. Overlap of the binary images of the novelty encoding effect for the three tasks showing common areas of activation. Neurological convention is used. Contrasts are overlaid over a canonical template with MNI coordinates at the bottom right of each slice. HP, hippocampus; PHG, parahippocampal gyrus; Fs, fusiform gyrus; Post, postcentral gyrus; IFGorb, inferior frontal gyrus pars orbitalis; IFGop, inferior frontal gyrus pars opercularis; MOG, middle occipital gyrus; IOG, inferior occipital gyrus; Prc, precentral gyrus; IFG, inferior frontal gyrus; ITG, inferior temporal gyrus; MTG, middle temporal gyrus; L, Left Hemisphere; R, Right Hemisphere.

Novel versus repeated stimuli contrasts (all conditions—W, F, S—pooled together) yielded robust activation in the HP, PHG and fusiform gyri bilaterally (see Table 2 and Fig. 4). However, this pattern of brain region activity seemed to emerge differently according to the stimulus condition presented during the encoding phase. Thus, novel versus repeated *word pairs* elicited enhanced neural activation in bilateral occipito-temporal cortex, left inferior frontal gyrus (IFG), left middle and inferior temporal gyrus and left PHG. The encoding of novel when compared to repeated *face-name associations* showed enhanced activity bilaterally in the HP, PHG and fusiform gyri and also in the left IFG and left middle and inferior temporal gyrus. Finally, the encoding of novel versus repeated pictures of *complex scenes* exhibited greater activation in the HP, PHG and fusiform gyrus bilaterally and showed massive activation in posterior parietal and occipital gyri (see S1 Table and Fig. 5A).

**Table 2:** Enhanced group-level fMRI-signal for the Novel > Repeated encoding trials for all three conditions pooled together (words, faces, scenes).

		Anatomical Region	BA	x	y	z	t-value	Cluster size
*All*	R	I/M/SOG; Fs; PHG; HP; I/SPG; I/MTG; Cerebellum; Lingual Gyrus	19,18,37,7	37.5	−85.5	3	12.46	28280
	L	I/M/SOG; Fs; PHG; HP; I/SPG; I/MTG; Thalamus; Putamen; Cerebellum; Lingual Gyrus; Precuneus	19,18,37,7	−39	−85.5	6	11.29	30765
	L	IFG orb/oper/tri; Prc; Insula	44,45,46,69,11	−34.5	34.5	−16.5	9.51	9602
	L	Orbitofrontal Cortex	11	−1.5	46.5	−21	5.71	1274
	R	IFG oper; Prc; Post	9,6	49.5	9	30	5.60	1958
	L	SMA	6	−3	19.5	52.5	5.22	2684
	R	Precuneus; Lingual Gyrus	30	11	−51	9	4.82	994
	R	IFG orb	11	33	36	−13.5	4.81	332
	R	IFG tri	46	46.5	31.5	15	4.73	548
	L	SFG	10	−6	64.5	33	3.66	121
	R	Prc	6	43.5	−6	63	3.45	75
	L	Caudate	25	−3	15	−7.5	3.36	270
	R	Putamen; Caudate	-	18	10.5	4.5	3.34	238
	L	Prc	6	− 28.5	0	63	3.34	232
	L	Post; IPG	40	−39	−33	49.5	3.14	165
	L	SFG	8	−15	42	54	3.07	102
	L	HP	−	−15	−31.5	−3	3.05	76

Results are shown at a p < 0.05 FDR-corrected threshold with 50 voxels of cluster extent. MNI coordinates are used. BA, Brodmann area; L, Left hemisphere; R, Right hemisphere.

IOF, inferior occipital gyrus; MOG, middle occipital gyrus; SOG, superior occipital gyrus; Fs, fusiform gyrus; HP, hippocampus; PHG, parahippocampal gyrus; HP, hippocampus; SPG, superior parietal gyrus; IPG, inferior parietal gyrus; ITG, inferior temporal gyrus; MTG, middle temporal gyrus; Post, postcentral gyrus; IFG orb, inferior frontal gyrus pars orbitalis; IFG oper, inferior frontal gyrus pars opercularis; IFG tri, inferior frontal gyrus pars triangularis; Prc, precentral gyrus; Post, postcentral gyrus; SMA, supplementary motor area; SFG; superior frontal gyrus

There was, however, partial overlap of brain activity across tasks depending on the type of material used, dividing them i nto verbal-material tasks (including words and face-name pairs) and non-verbal-material tasks (including complex visual scenes and face-name pairs; see Fig. 5B). The encoding of novel verbal-material induced an activity enhancement of left-lateralized brain areas: IFG, middle frontal gyrus (MFG), fusiform gyrus and PHG. In contrast, the encoding of novel nonverbal-material engaged greater activity at the HP, PHG and fusiform gyrus bilaterally.

**Fig 6:**
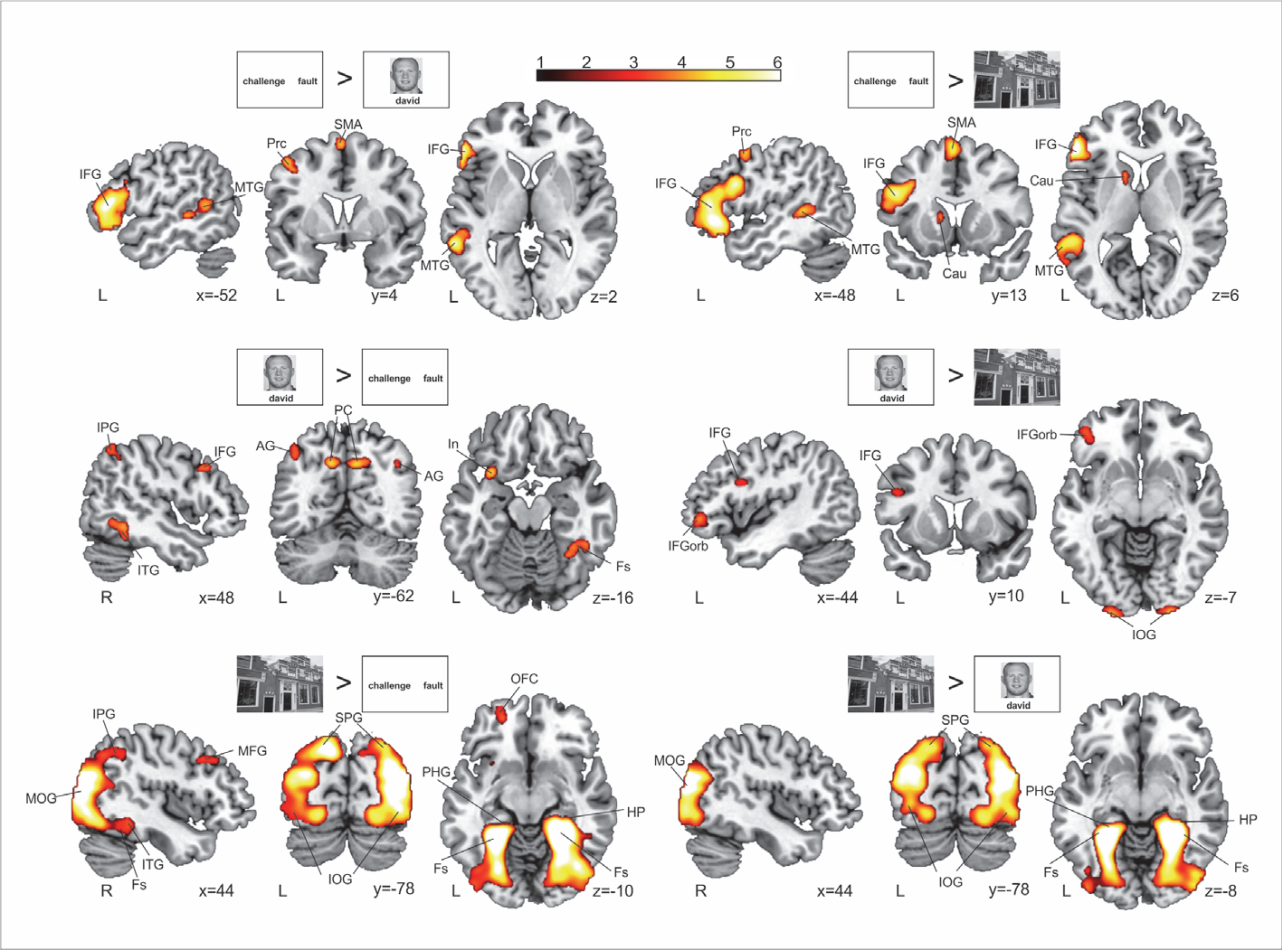
Whole brain between-material differences in the novelty contrast. In red-yellow, enhanced group-level fMRI-signal for the Novel > Repeated encoding trials for words > faces (first row, left), words > scenes (first row, right), faces > words (second row, left), faces > scenes (second row, right), scenes > words (third row, left), scenes > faces (third row, right). All statistical maps are thresholded at p < 0.05 FDR-corrected with 50 voxels of cluster extent. Neurological convention is used. Contrasts are overlaid over a canonical template with MNI coordinates at the bottom right of each slice. Prc, precentral gyrus; IFG, inferior frontal gyrus; MTG, middle temporal gyrus; SMA, supplementary motor area; IPG, inferior parietal gyrus; ITG, inferior temporal gyrus; AG, angular gyrus; PC, precuneus; In, insula; Fs, fusiform gyrus; MOG, middle occipital gyrus; IOG, inferior occipital gyrus; MFG, middle frontal gyrus; SPG, superior parietal gyrus; OFC, orbitofrontal cortex; HP, hippocampus; PHG, parahippocampal gyrus; IFGorb, inferior frontal gyrus pars orbitalis; Cau, Caudate; L, Left Hemisphere; R, Right Hemisphere.

This pattern of activation is further supported by the results provided by the comparison of the Novelty > Repeated contrast between conditions (see Fig. 6 and S2 Table). Specifically, word pairs elicited enhanced fMRI activity in left lateralized inferior frontal and middle temporal regions when compared to face-name pairs and scenes. Interestingly, face-name pairs also elicited more activity in the left IFG than scenes. On the other hand, when compared with words, face name pairs activated more right lateralized inferior parietal, temporal and frontal areas and the fusiform gyrus. Finally, scenes showed strong effects bilaterally in posterior parietal and occipital areas and in the fusiform and parahippocampal gyri when compared with both face-name and word pairs (although the differences where stronger and more right lateralized for the comparison against words).

## Discussion

The present study shows that activity from the HP and surrounding MTL structures, such as PHG, can be reliably identified at the individual level on the basis of a novel fMRI-based task design that promoted novelty detection. In addition, current design induced HP and PHG activity at the individual level in a high percentage of participants independent of the type of associative novelties among previously studied material (see Fig. 2C and 2D), thereby contributing to the idea that the underlying mechanism of novelty detection is non-domain specific [3,18–24]. The possibility of studying MTL activity at the individual level represents a hallmark in clinical settings. We believe our task design may also be useful in a broad range of translational research targeting MTL activity. Although there is a prominent consensus on the general role of the MTL in episodic memory functioning [41], fMRI studies have also shown that the detection of activity within the MTL—and more concretely from the HP—is technically challenging [13]. Here, we overcame this limitation by using a ROI approach centered at the HP and the PHG, thereby enhancing the sensitivity of our analyses [16,17].

We believe that the need to encode arbitrary associations among presented elements in all encoding conditions promoted HP processing, and therefore, its identification at the fMRI level. Interestingly, it is known from theoretical and human lesion studies that relational processing is a fundamental operation in the HP [5,6,37,42,43]. We further potentiated HP functioning by contrasting activity induced in trials involving the detection of novel compared to familiar relationships. Thus, given that novelty detection has been shown to be computed mainly in the HP, our task promotes even further the recruitment of this structure. Previous fMRI studies showed that the HP responded to the novelty of spatial and non-spatial object relationships but not to the novelty of individual objects, which selectively activated PHG structures only (i.e., the perirhinal cortex [24]). Notably, our findings extend this past research by revealing that HP activity is induced by the detection of novel relationships to other sorts of material such as complex scenes, words and face-name pairs at the individual level. In addition, the present HP results also support the notion that functions of the human HP are not limited to the spatial domain [44].

An additional advantage of our task design is that it also allowed investigating possible laterality effects as a function of stimulus material: our HP ROI analysis showed a stimuli-dependent lateralization, left lateralized for word encoding and bilateral for face-word and scene encoding (see Fig. 2B). These results support the notion of the existence of brain laterality effects due to the type of stimulus material [45]. Indeed, our present investigation follows the lines of previous studies which showed that the encoding process is determined by the verbalizability of the stimuli being encoded [3]: left-lateralized for word, bilateral for picture and right-lateralized for face encoding [46].

Additionally, the current ROI-based analysis in combination with our task design showed that all encoding conditions activated bilateral PHG in up to 80% of participants. However, among the three conditions, the encoding of novel complex visual scenes was the one that showed the most consistent activation across participants (100% of the participants showed clear activation in the right PHG, see Fig. 2D). Notably, our findings reveal differences in the response profile of different portions of the PHG along its longitudinal axis. Thus, while the encoding of novel scenes showed a high pattern of activity in the posterior part of the PHG, word and face-name novel pair encoding activated more anterior regions of the PHG, including the perirhinal and entorhinal cortex (see Fig. 2C). These results are in line with previous studies reporting that the PHG contains functionally distinct regions, with the posterior parahippocampal cortex preferentially supporting scene encoding [47]. However, both our word-pair and face-name tasks clearly engaged the PHG. This may be explained by the fact that in the present investigation both the word-pair and face-name conditions comprised an associative encoding task that possibly contributed to the engagement of more anterior regions of the PHG [47].

The possibility of studying brain responses to novel relationships outside the MTL structures (during the group whole brain analyses, see Fig. 4, 5 and 6) adds a valuable contribution on how novelty responses emerge in the brain. Thus, most of the previous fMRI studies have focused mainly on the analysis of MTL structures, leaving out the possibility of investigating how such MTL activity acts in coordination with activity from other cortical regions. However, most of the influential theoretical models on how new memories are formed in the brain agree in suggesting that the MTL, and the HP more concretely, acts coordinately with neocortical regions [48–51]. Thus, although our data do not contribute directly to questioning how these two learning systems (i.e., HP and neocortical) cooperate during encoding—which would require for instance functional connectivity analyses [52,53]—they still show the selective recruitment of neocortical regions as a function of the established relation among elements at encoding. Indeed, the whole brain analysis of the novelty effect at the group level showed that verbal tasks, including the word pair and the face-name associations, overlapped in left anterior regions such as the middle frontal gyrus and IFG. On the other hand, non-verbal tasks, including the complex visual scenes and the face-name conditions, overlapped bilaterally in more posterior regions such as the fusiform gyri and the inferior and middle occipital gyri (see Fig. 5 and 6).

In summary, our fMRI task design represents a reliable approach to the study of brain activity implicated in novelty. More importantly, it provides a means to track MTL activity at the individual level, focusing on functional activity of the HP and the PHG, and thus providing a useful tool for future research in clinical memory-related fMRI studies.

## Supporting Information

S1 Table Enhanced group-level fMRI-signal for the Novel > Repeated encoding trials for words, faces and scenes. Results are shown at a p < 0.05 FDR-corrected threshold with 50 voxels of cluster extent. MNI coordinates are used. BA, Brodmann area; L, Left hemisphere; R, Right hemisphere.
(DOCX)

S2 Table Enhanced group-level fMRI-signal for the differences in the Novel > Repeated contrast between words, faces and scenes. Results are shown at a p < 0.05 FDR-corrected threshold with 50 voxels of cluster extent. MNI coordinates are used. BA, Brodmann area; SMA, Supplementary Motor Area; L, Left; R, Right; B, Bilateral; W, Words; F, Faces; S, Scenes.
(DOCX)
