# Supplementary material for: Studying Memory Encoding to Promote Reliable Engagement of the Medial Temporal Lobe at the Single-Subject Level
Source: PLoS One. 2015 Mar 24;10(3):e0119159. doi: 10.1371/journal.pone.0119159 (PMC4372361; doi:10.1371/journal.pone.0119159)
Supplement: S1 Table — Results are shown at a p < 0.05 FDR-corrected threshold with 50 voxels of cluster extent. MNI coordinates are used. BA, Brodmann area; L, Left hemisphere; R, Right hemisphere. (DOCX) [file pone.0119159.s001.docx]

**S1 Table** Enhanced group-level fMRI-signal for the Novel > Repeated encoding trials for words, faces and scenes. Results are shown at a p < 0.05 FDR-corrected threshold with 50 voxels of cluster extent. MNI coordinates are used. BA, Brodmann area; L, Left hemisphere; R, Right hemisphere.

|  |  | **Anatomical Region** | **BA** | **x** | **y** | **z** | **t-value** | **Cluster size** |
| --- | --- | --- | --- | --- | --- | --- | --- | --- |
| *Words* | L | IFG orb/oper/tri; Prc; MFG | 44,45,46,69,11 | -34.5 | 33 | -18 | 9.75 | 10029 |
|  | L | SMA; SFG | 6,8,9,10 | -3 | 13.5 | 66 | 6.32 | 4499 |
|  | L | I/MTG; Fs; PHG; I/MOG; Cerebellum | 37,18,19 20,22 | -45 | -54 | -15 | 6.09 | 9048 |
|  | R | Cerebellum; I/MOG | 18,19 | 10.5 | -76.5 | -28.5 | 5.48 | 6140 |
|  | L | Orbitofrontal Cortex | 11 | -1.5 | 46.5 | -19.5 | 4.78 | 783 |
|  | R | Caudate; Putamen | - | 16.5 | 7.5 | 6 | 4.69 | 742 |
|  | L | Caudate; Putamen | - | -13.5 | 10.5 | 7.5 | 4.36 | 566 |
|  | L | Thalamus | - | -7.5 | -16.5 | 9 | 4.19 | 320 |
|  | R | IFG tri/oper; Prc | 45,46,6 | 61.5 | 16.5 | 24 | 4.01 | 632 |
|  | L | Precuneus | 30 | -4.5 | -52.5 | 13.5 | 3.93 | 334 |
|  | L | Angular Gyrus | 39 | -48 | -69 | 27 | 3.81 | 140 |
| *Faces* | R | I/MTG; I/M/SOG; Fs; PHG; Cerebellum; Lingual Gyrus | 18,19,20 37,39 | 45 | -66 | -12 | 8,42 | 11482 |
|  | L | IFG oper/tri; Prc | 6,9,45 | -46,5 | 7,5 | 30 | 6,79 | 2544 |
|  | L | I/M/SOG; I/MTG; Fs; PHG; Cerebellum; Lingual Gyrus; HP | 18,19,20 37 | -36 | -79,5 | -10,5 | 6,59 | 8354 |
|  | R | IFG oper; Prc | 9 | 49,5 | 9 | 28,5 | 6,11 | 847 |
|  | L | IFG orb | 11 | -33 | 34,5 | -16,5 | 5,85 | 621 |
|  | L | Orbitofrontal Cortex | 11 | 0 | 45 | -19,5 | 4,45 | 768 |
|  | R | MFG; IFG tri | 46 | 45 | 31,5 | 19,5 | 4,25 | 467 |
|  | R | Post | 4,6 | 16,5 | -34,5 | 58,5 | 4,14 | 260 |
|  | L | I/SPG | 7, 40 | -28,5 | -48 | 48 | 4,09 | 783 |
|  | R | I/SPG | 7, 40 | 27 | -54 | 52,5 | 3,98 | 947 |
|  | L | SMA | 6,8 | 0 | 12 | 54 | 3,87 | 346 |
|  | L | Putamen | - | -22,5 | 1,5 | -7,5 | 3,67 | 195 |
|  | R | HP; PHG | - | 21 | -10,5 | -16,5 | 3,50 | 97 |
|  | R | HP; PHG | - | 34,5 | -10,5 | -24 | 3,37 | 87 |
|  | R | Thalamus |  | 4,5 | -9 | 0 | 3,31 | 88 |
|  | R | IFG orb | 11 | 33 | 36 | -12 | 3,30 | 78 |
|  | L | HP | - | -16,5 | -9 | -21 | 3,29 | 59 |
| *Scenes* | L/R | Fs; I/M/SOG; I/MTG; PHG; HP; I/SPG; Lingual Gyrus; Precuneus; Cuneus; Calcarine; Thalamus; Cerebellum | 7,18,19,20,30,35,36,37,39 | -27 | -45 | -9 | 16.12 | 61027 |
|  | R | IFG orb | 11 | 31.5 | 36 | -12 | 4.54 | 269 |
|  | R | IFG oper; Prc | 9 | 39 | 9 | 28.5 | 4.20 | 739 |
|  | L | IFG orb | 11 | -34.5 | 36 | -16.5 | 3.90 | 149 |
|  | L | IFG oper; Prc | 9 | -33 | 13.5 | 27 | 3.79 | 381 |
|  | L | Globus Pallidum; Putamen | - | -21 | -4.5 | 0 | 3.76 | 179 |
|  | R | Prc | 6 | 54 | -1.5 | 46.5 | 3.68 | 372 |
|  | R | HP; Amygdala | - | 30 | -4.5 | -19.5 | 3.40 | 79 |
|  | R | Post | 3 | 42 | -24 | 43.5 | 3.19 | 133 |
| IFG orb, inferior frontal gyrus pars orbitalis; IFG oper, inferior frontal gyrus pars opercularis; IFG tri, inferior frontal gyrus pars triangularis; Prc, precentral gyrus; MFG, middle frontal gyrus; SMA, supplementary motor area; SFG; superior frontal gyrus; ITG, inferior temporal gyrus; MTG, middle temporal gyrus; Fs, fusiform gyrus; HP, hippocampus; PHG, parahippocampal gyrus; IOF, inferior occipital gyrus; MOG, middle occipital gyrus; SOG, superior occipital gyrus; SPG, superior parietal gyrus; IPG, inferior parietal gyrus; Post, postcentral gyrus | | | | | | | | |
