# Supplementary material for: Studying Memory Encoding to Promote Reliable Engagement of the Medial Temporal Lobe at the Single-Subject Level
Source: PLoS One. 2015 Mar 24;10(3):e0119159. doi: 10.1371/journal.pone.0119159 (PMC4372361; doi:10.1371/journal.pone.0119159)
Supplement: S2 Table — Results are shown at a p < 0.05 FDR-corrected threshold with 50 voxels of cluster extent. MNI coordinates are used. BA, Brodmann area; SMA, Supplementary Motor Area; L, Left; R, Right; B, Bilateral; W, Words; F, Faces; S, Scenes. (DOCX) [file pone.0119159.s002.docx]

**S2 Table** Enhanced group-level fMRI-signal for the differences in the Novel > Repeated contrast between words, faces and scenes. Results are shown at a p < 0.05 FDR-corrected threshold with 50 voxels of cluster extent. MNI coordinates are used. BA, Brodmann area; SMA, Supplementary Motor Area; L, Left; R, Right; B, Bilateral; W, Words; F, Faces; S, Scenes.

|  | **Anatomical Region** | **BA** | **x** | **y** | **z** | **t-value** | **Cluster** |
| --- | --- | --- | --- | --- | --- | --- | --- |
| *W>F* | L Inferior Frontal Gyrus | 44,45,46,47 | -56 | 27 | 10 | 6.95 | 2101 |
|  | L Mid./Sup. Temporal Gyrus | 21,22 | -60 | -43 | 1 | 5.99 | 913 |
|  | L Precentral Gyrus | 6 | -45 | 8 | 51 | 4.64 | 302 |
|  | L SMA Area; L Superior Medial Frontal Gyrus | 6,8 | -3 | 29 | 60 | 4.62 | 506 |
|  | L Superior Medial Frontal Gyrus | 8,9 | -6 | 54 | 46 | 4.35 | 268 |
| *W>S* | L Inf./Mid. Frontal Gyrus; L Precentral Gyrus | 9,11,44,45  46,47 | -53 | 35 | 4 | 7.79 | 5274 |
|  | L Mid./Sup. Temporal Gyrus | 21,22 | -59 | -42 | 3 | 5.75 | 1067 |
|  | B SMA Area; B Superior Medial Frontal Gyrus | 6,8 | -3 | 26 | 57 | 5.31 | 1677 |
|  | L Superior Frontal Gyrus | 9,10 | -9 | 62 | 27 | 4.92 | 473 |
|  | L Precentral Gyrus | 6 | -45 | 6 | 48 | 4.54 | 259 |
|  | R Inferior Occipital Gyrus | 18 | 26 | -100 | -6 | 4.53 | 243 |
|  | R Inferior Frontal Gyrus | 45,47 | 56 | 35 | 0 | 4.25 | 86 |
|  | R Cerebellum | - | 17 | -76 | -30 | 4.20 | 239 |
|  | L Inferior Occipital Gyrus | 18 | -23 | -104 | -8 | 3.94 | 216 |
|  | L Caudate | - | -12 | 11 | 8 | 3.88 | 146 |
| *F>W* | B Precuneus; B Cuneus; L Sup. Parietal Gyrus; L Sup. Occipital Gyrus | 7 | -9 | -73 | 43 | 5.57 | 1530 |
|  | L Insula | 13 | -32 | 12 | -15 | 4.60 | 151 |
|  | L Inferior Parietal Gyrus; L Angular Gyrus | 40 | -42 | -54 | 46 | 4.43 | 608 |
|  | L Orbitofrontal Cortex | 11 | -21 | 50 | -9 | 4.35 | 152 |
|  | R Fusiform Gyrus; R Inferior Temporal Gyrus | 19,37 | 48 | -46 | -11 | 4.12 | 613 |
|  | R Inferior Parietal Gyrus; R Angular Gyrus | 40 | 41 | -48 | 42 | 3.96 | 454 |
|  | R Middle Frontal Gyrus | 9 | 44 | 23 | 36 | 3.80 | 240 |
| F>S | R Inf. occipital Gyrus; R Calcarine; R Lingual Gyrus | 17,18 | 26 | -100 | -6 | 4.45 | 323 |
|  | L Inf. occipital Gyrus; L Calcarine; L Lingual Gyrus | 17,18 | -23 | -102 | -11 | 4.25 | 499 |
|  | L Inferior Frontal Gyrus | 47 | -44 | 42 | -3 | 3.90 | 347 |
|  | L Precentral Gyrus | 9 | -47 | 9 | 27 | 3.62 | 136 |
| S>W | B Mid./Sup/Inf. Occipital Gyrus; B Fusiform Gyrus; B Precuneus; B Sup./Inf. Parietal Gyrus; B Lingual Gyrus; R Mid./Inf. Temporal Gyrus; B Angular Gyrus; B Mid. Cingulate Gyrus;  B Parahippocampal Gyrus; B Cuneus; B Cerebellum; B Calcarine; R Hippocampus | 7,18,19,20,23,30,31,36,37,39,40 | 27 | -43 | -8 | 13.23 | 46000 |
|  | L Orbitofrontal Cortex | 11 | -21 | 51 | -12 | 3.81 | 228 |
|  | R Mid./Sup Frontal gyrus | 8 | 21 | 26 | 45 | 3.78 | 453 |
|  | L Insula | 13 | -30 | 14 | -14 | 3.76 | 109 |
|  | R Middle Frontal Gyrus | 9 | 48 | 17 | 39 | 3.68 | 345 |
|  | L Superior Frontal Gyrus | 10 | -21 | 63 | 6 | 3.57 | 390 |
| S>F | L Inf./Mid./Sup. Occipital Gyrus; L Fusiform Gyrus; L Sup./Inf. Parietal Gyrus; L Lingual Gyrus; L Cuneus; L Precuneus; L Parahippocampal Gyrus; L Calcarine; L Cerebellum; L Mid. Temporal Gyrus; L Hippocampus | 7,18,19,21,30,31,36,37,39 | -27 | -45 | -9 | 13.65 | 15182 |
|  | R Inf./Mid./Sup Occipital Gyrus; R Fusiform Gyrus; R Lingual Gyrus; R Precuneus; R Parahippocampal Gyrus; R Sup. Parietal Gyrus; R Inf./Mid. Temporal Gyrus; R Calcarine; R cerebellum; R Cuneus; R Angular Gyrus; R Hippocampus; R Posterior Cingulate Gyrus | 7,18,19,20,2129,30,31,35 36,37,39 | 26 | -43 | -8 | 12.43 | 18308 |
|  | R Precuneus; R Middle Cingulate Gyrus | 7,31 | 11 | -43 | 46 | 3.51 | 99 |
|  | R Sup./Mid. Frontal Gyrus | 8 | 21 | 27 | 42 | 3.50 | 110 |
